# Differentiating Co-Delivery of Bisphosphonate and Simvastatin by Self-Healing Hyaluronan Hydrogel Formed by Orthogonal “Clicks”: An In-Vitro Assessment

**DOI:** 10.3390/polym13132106

**Published:** 2021-06-26

**Authors:** Dmitri A. Ossipov, Mads Lüchow, Michael Malkoch

**Affiliations:** 1 Department of Biosciences and Nutrition, Karolinska Institutet, SE-141 83 Stockholm, Sweden; 2 Department of Fiber and Polymer Technology, KTH Royal Institute of Technology, SE-100 44 Stockholm, Sweden; luchow@kth.se (M.L.); malkoch@kth.se (M.M.)

**Keywords:** hyaluronan, bisphosphonate, prodrug, simvastatin, hydrogel, orthogonal reactions

## Abstract

Due to its unique properties resembling living tissues, hydrogels are attractive carriers for the localized and targeted delivery of various drugs. Drug release kinetics from hydrogels are commonly controlled by network properties and the drug-network interactions. However, and simultaneously, the programmable delivery of multiple drugs with opposing properties (hydrophilicity, molecular weight, etc.) from hydrogels with determined network properties is still challenging. Herein, we describe the preparation of injectable self-healing hyaluronic acid (HA) hydrogels that release hydrophobic simvastatin and hydrophilic aminobisphosphonate (BP) drugs independently in response to acidic and thiol-containing microenvironments, respectively. We apply a prodrug strategy to BP by conjugating it to HA via a self-immolative disulfide linker that is stable in the blood plasma and is cleavable in the cytoplasm. Moreover, we utilize HA-linked BP ligands to reversibly bind Ca^2+^ ions and form coordination hydrogels. Hydrazone coupling of hydrophobic ligands to HA permits the encapsulation of simvastatin molecules in the resulting amphiphilic HA derivative and the subsequent acid-triggered release of the drug. The conjugation of BP and hydrophobic ligands to HA enables preparation of both bulk self-healing hydrogels and nanogels. Moreover, the developed hydrogel system is shown to be multi-responsive by applying orthogonally cleavable linkers. The presented hydrogel is a potential candidate for the combination treatment of osteoporosis and bone cancers as well as for bone tissue regeneration since it can deliver bone anabolic and anti-catabolic agents in response to bone diseases microenvironments.

## 1. Introduction

In the treatment of bone diseases, it is desirable to provide both bone anabolic and anti-catabolic treatments. For example, in bone cancer that occurs after the metastasizing of a primary tumor [1], cancer cells and bone-resorbing cells (osteoclasts) act in concert [2], and it is recommended to augment chemotherapy with anti-osteoclastic drugs that prevent bone resorption [3]. Aminobisphosphonates and estrogens have a significant effect on bone remodeling by inhibiting bone breakdown and, thus, represent the main medications for the treatment of osteoporosis [4]. Recently, it was also proposed to treat osteoporosis with bone anabolic agents that increase bone mass in contrast to anti-osteoclastic drugs, the prolonged intake of which lower bone turnover [5]. A combined therapy of bisphosphonates with agents that stimulate osteoblastic function should greatly increase bone mass and prevent osteoporotic fractures [6,7,8]. However, bisphosphonates (BPs) are highly hydrophilic molecules, whereas most anti-cancer drugs, estrogens, and agents with bone anabolic effect such as statins and hormone prostaglandin E_2_ (PGE_2_) are poorly soluble in water. The co-delivery of drugs with such opposing physical properties with controlled release kinetics is a challenge.

Hydrogels are a particularly attractive class of biomaterials for the controlled delivery of various drugs due to their tunable physical properties, programmed degradability, and ability to protect labile drugs, properties that can be controlled through the molecular design of polymer networks [9,10,11].

Bisphosphonates were known as anti-osteoporotic drugs for more than forty years based on their suppressing effect on bone resorbing cells, osteoclasts [12]. However, bisphosphonates fail to restore the initial bone density and architecture since, at best, they stop bone resorption but cannot stimulate bone growth. On the other hand, statins, inhibitors of 3-hydroxy-3-methylglutaryl-coenzyme reductase, were reported in several studies to induce an increase of bone mineral density (BMD) [13,14] through inhibiting the expression of matrix metalloproteases and promoting osteoblast differentiation [15]. This bone anabolic effect of statins, however, was not shown in some other in vivo studies, which was explained by poor aqueous solubility of statins and their low concentration at the bone site [16]. Based on very high affinity of BPs to the hydroxyapatite mineral part of bone, they were also utilized as bone targeting groups in the conjugation of BPs to different molecules (polymers, proteins, low molecular weight therapeutics) and nanoparticulate carriers for drugs [17]. Considering the perspectives concerning the combination of anti-resorptive and bone-inducing agents, nanotechnology-based bone targeting drug delivery systems are urgently needed.

Hyaluronic acid (HA) is a natural glycosaminoglycan which is found in connective tissues and plays an important role as a signaling molecule in cell proliferation and motility, extracellular matrix organization, and morphogenesis [18]. Due to its biodegradability, biocompatibility, and non-immunogenicity, HA hydrogels were studied as localized and systemic drug delivery vehicles [19] for a variety of medical applications including bone tissue engineering [20], the curing of osteoporosis [7,8], and treatment of bone cancer [21].

**Figure 1:**
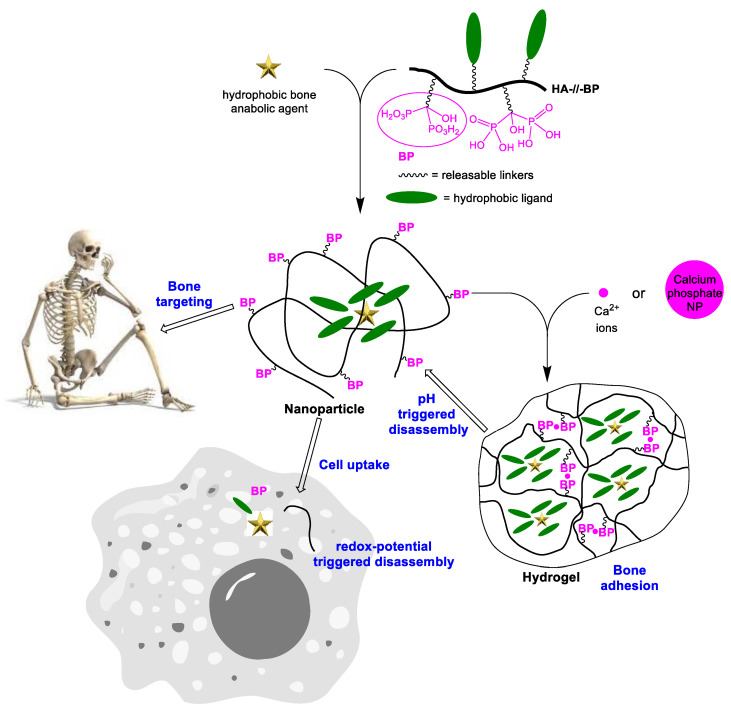
The nanomedicine platform based on the self-assembly of hyaluronic acid conjugated to bisphosphonates and hydrophobic ligands through orthogonally releasable linkers. Hydrophobic encapsulation of bone anabolic agents occurs during the self-assembly and provide nanoparticles with a dual mode of bioactivity.

To address unmet needs in nanotechnology-based drug delivery vehicles for bone diseases, we sought to make aminobisphosphonates acting simultaneously for three important purposes: (i) to act as bone targeting groups; (ii) to be released intact and act as anti-osteoclastic/anti-cancer drugs; and (iii) to enable immobilization of anabolic bone agents into localized hydrogel biomaterials for their subsequent sustained release at the bone resorbing sites. Most of the studies concerned with the targeting of nanomedicines to bone have explored covalent attachment of BP ligands without mechanisms of their intact release. To the best of our knowledge, there have been only few reports on aminobisphosphonate conjugation via releasable intact linkages [22,23]. Here, we report on the development of a hydrogel platform for the bone-targeted delivery of aminobisphosphonates and statins based on a combination of prodrug strategy and physical encapsulation of hydrophobic drugs (Figure 1). To achieve this, we conjugated pamidronate (amino-BP) and hydrophobic ligands to the hydrophilic backbone of hyaluronic acid using chemoselective orthogonal reactions. Conjugation of hydrophilic amino-BP and hydrophobic ligands to HA provides self-assembling properties of the resulting conjugate and enables encapsulation of hydrophobic cargos in the core of the generated nanoparticles. Moreover, orthogonal conjugation chemistry ensures the differential release of the drugs. For the first time, we designed the release of aminobisphosphonates intact in the cytoplasmic microenvironment and the release of simvastatin under acidic conditions that are characteristic for the bone resorption sites. Moreover, we utilize coordination binding of the attached BPs to metal cations, metal oxides, and inorganic salts nanoparticles to transform the nanoparticles into self-healing bulk hydrogels and show that the hydrogels can disassemble under acidic and reducing conditions. Our modular assembly approach, based on “clickable” chemical reactions and orthogonal release mechanisms, is generic and can be extended to a broad range of anti-cancer drugs and metal ions/coordination nanoparticles with multifunctional magnetic, electrical, and imaging properties.

## 2. Materials and Methods

Hyaluronic acid (HA) sodium salt (MW 150 kDa) was purchased from Lifecore Biomedical (Chaska, MN, USA). 1-Ethyl-3-(3-dimethylaminopropyl) carbodiimide (EDC) and *N*-hydroxybenzotriazole (HOBt) were purchased from Fluka (Buchs, Switzerland). DL-dithiothreitol (DTT) was purchased from Aldrich Chemical Co (St. Louis, MO, UAS). *N*,*N*′-disuccinimidyl carbonate (DSC) and simvastatin (SIM) was purchased from ACROS Organics ™ (Fair Lawn, NJ, USA). 2-mercaptopyridine and 1-fluoro-2,4-dinitrobenzene were purchased from Alfa Aesar ™. 2-hydroxy-1-methyl-ethyl mercaptan **1** [24] and *N*-(6-aminohexyl)-2,4-dinitroaniline hydrochloride [25] were synthesized according to the literature. Aldehyde-modified hyaluronic acid (HA-al) [26], thiol-modified hyaluronic acid (HA-SH) [26], HA dually modified with thiol and hydrazide groups (hy-HA-SH) [27], and HA dually modified with bisphosphonate and thiol groups (BP-HA-SH) [28] were synthesized according to our previously published protocols. All solvents were of analytical quality (p.a.) and were dried over 4Å molecular sieves. Dialysis membranes Spectra/Por 6 (3500 g/mol cutoff) were purchased from VWR international. ^1^H-NMR spectra were recorded in D_2_O with a Bruker NMR spectrometer at a magnetic field strength 9.4 T, operating at 400 MHz. UV–Vis absorption spectra were recorded using UV–Vis spectrometer (Cary 300 Bio from Varian).

### 2.1. Synthesis of 2-(2-Pyridyldithio)-2-Methylethanol 2

2-mercaptopyridine (504.4 mg, 4.538 mmol) was dissolved in dry dichloromethane (5 mL) and cooled down to 0–5 °C. A solution of sulfonyl chloride (680 mg, 5.037 mmol) in dry DCM (5 mL) was added dropwise over a period of 8 min to the stirred and cooled solution of 2-mercaptopyridine. Yellow precipitate was formed and the mixture was stirred at room temperature in a nitrogen atmosphere for 2.5 h. The mixture was concentrated on a rotary evaporator and the solid yellow residue was resuspended in 5 mL of dry DCM. The suspension was cooled down on an ice bath and a solution of 2-hydroxy-1-methyl-ethyl mercaptan **1** (417.5 mg, 4.538 mmol) in dry DCM (2 mL) was subsequently added to the stirred and cold suspension in two portions over a period of 5 min. The resulting mixture was stirred at room temperature overnight. After the reaction, the mixture was evaporated, re-suspended in 3 mL of DCM, and treated with 4-(dimethylamino)pyridine (582 mg, 4.765 mmol). The deprotonated product was purified by silica gel column chromatography using 0–5% ethanol in dichloromethane as an eluent. Yield—848 mg (92.9%). ^1^H-NMR (CDCl_3_): 8.51 (1H, d, 2-pyridyl, *J* = 5.0 Hz), 7.59 (1H, td, 2-pyridyl, *J* = 8.0 Hz, *J* = 1.9 Hz), 7.41 (1H, d, 2-pyridyl, *J* = 8.0 Hz), 7.17 (1H, m, 2-pyridyl), 3.70 (1H, d, –CH_2_–, *J* = 10.4 Hz), 3.41 (1H, dd, –CH_2_–, *J*_gem_ = 12.3 Hz, *J*_vic_ = 7.9 Hz), 3.12 (1H, m, –CH<), 1.32 (3H, d, methyl, *J* = 7 Hz).

### 2.2. Synthesis of 2-(2-Pyridyldithio)-2-Methylethyl N-Hydroxysuccinimide Carbonate 3

2-(2-pyridyldithio)-2-methylethanol **2** (215 mg, 1.07 mmol) was dissolved in dry acetonitrile (11 mL). *N*, *N*′-disuccinimidyl carbonate (548.2 mg, 2.14 mmol) was dissolved in a mixture of dry acetonitrile (9 mL), dry pyridine (1 mL), and triethylamine (216.1 mg, 2.14 mmol). DSC solution was added to the solution of **1** under stirring and nitrogen atmosphere. The resulting reaction mixture was stirred at room temperature in a nitrogen atmosphere for 24 h. The mixture was evaporated to dryness and the residue was purified by silica gel column flash chromatography using 0–10% ethyl acetate in dichloromethane as an eluent. Yield—276 mg (75.4%). ^1^H-NMR (CDCl_3_): 8.50 (1H, d, 2-pyridyl, *J* = 4.8 Hz), 7.70 (2H, d, 2-pyridyl, *J* = 4.4 Hz), 7.13 (1H, q, 2-pyridyl, *J* = 4.6 Hz), 4.50 (1H, dd, –CH_2_–, *J*_gem_ = 10.9 Hz, *J*_vic_ = 5.6 Hz), 4.33 (1H, dd, –CH_2_–, *J*_gem_ = 10.9 Hz, *J*_vic_ = 5.6 Hz), 3.33 (1H, m, –CH<), 2.86 (4H, s, succinimide), 1.43 (3H, d, methyl, *J* = 7.0 Hz).

### 2.3. Synthesis of Releasable 2-Pyridyldithio Derivative of Pamidronate 4

Pamidronate hydrochloride (66 mg, 0.243 mmol) was re-suspended in 1.4 mL of water and triethylamine (103 µL, 0.74 mmol) was added to the suspension to solubilize the starting bisphosphonate. Carbonate **3** (141.2 mg, 0.4126 mmol) was separately dissolved in acetonitrile (2 mL) and the pamidronate solution was added dropwise to the solution of carbonate **3** under stirring. The obtained clear reaction solution was stirred for 20 h and then concentrated on a rotary evaporator to remove acetonitrile. The concentrated turbid aqueous solution was washed with diethyl ether (3 × 7 mL) and the cleared aqueous phase was separated and evaporated to dryness. The oily residue was washed with acetone, and the formed precipitate was isolated by centrifugation and dried. Yield—140.5 mg (75.3%). ^1^H-NMR (D_2_O): 8.26 (1H, d, 2-pyridyl), 7.82 (2H, d, 2-pyridyl), 7.25 (1H, q, 2-pyridyl), 4.00–3.80 (2H, m, –CH(CH_3_)CH_2_–), 3.26–3.19 (3H, m, –CH< and –NHCH_2_–), 3.09 (18H, q, –CH_2_– from three Et_3_NH^+^ cations), 2.00 (2H, m, –CH_2_CH(PO_3_^2-^)_2_), 1.21 (3H, d, methyl), 1.18 (27H, t, –CH_3_ from three Et_3_NH^+^ cations).

### 2.4. Synthesis of 2-Pyridyldithio Derivative of N-(6-Aminohexyl)-2,4-Dinitroaniline 5

*N*-(6-aminohexyl)-2,4-dinitroaniline hydrochloride (174 mg, 0.51 mmol) dissolved in a mixture of dry acetonitrile (2 mL), dry pyridine (2 mL), and triethylamine (210 µL, 1.5 mmol). Carbonate **3** (102.7 mg, 0.3 mmol) was separately dissolved in dry acetonitrile (2 mL) and was mixed with the solution of the amine under stirring. The mixture was stirred under a nitrogen atmosphere and room temperature for 6 h, evaporated, and co-evaporated with toluene. The crude material was separated by silica gel column flash chromatography using 20% ethyl acetate in dichloromethane as an eluent. Yield—128.3 mg (80%). ^1^H-NMR (CDCl_3_): 9.16 (1H, d, 2,4-dinitroaniline residue, *J* = 2.5 Hz), 8.57 (1H, m, –C(O)NH–), 8.46 (1H, d, 2-pyridyl, *J* = 4.1 Hz), 8.29 (1H, dd, 2,4-dinitroaniline residue, ^2^*J* = 9.4 Hz, ^3^*J* = 2.5 Hz), 7.74 (1H, d, 2-pyridyl, *J* = 8.1 Hz), 7.65 (1H, td, 2-pyridyl, ^2^*J* = 7.5 Hz, ^3^*J* = 1.7 Hz), 7.10 (1H, dd, 2-pyridyl, ^2^*J* = 7.5 Hz, ^3^*J* = 4.7 Hz), 6.93 (1H, d, 2,4-dinitroaniline residue, *J* = 9.4 Hz), 4.74 (1H, m, –NH–), 4.22–4.16 (2H, m, –CH(CH_3_)CH_2_–), 3.43 (2H, q, –C(O)NHCH_2_–, *J* = 7.1 Hz), 3.26–3.17 (3H, m, –CH< and –CH_2_NH–), 1.79 (2H, quintet, –C(O)NHCH_2_CH_2_–, *J* = 7.2 Hz), 1.58–1.39 (6H, m, –CH_2_CH_2_CH_2_–), 1.35 (3H, d, methyl, *J* = 7.0 Hz).

### 2.5. Synthesis of Aldehyde-Modified DN Derivative 6

*N*-(6-aminohexyl)-2,4-dinitroaniline (DN, 303.2 mg, 0.89 mmol) was dissolved in a mixture of dry pyridine and dry acetonitrile (7 mL, 1:1 *v/v*) in the presence of triethylamine (410 µL, 2.965 mmol). *para*-formylbenzoic acid *N*-hydroxysuccinimide ester (146.6 mg, 0.593 mmol) was separately dissolved in dry acetonitrile (3.5 mL), and the obtained solution was added dropwise into the stirred solution of DN over 5 min. The obtained reaction solution was stirred under nitrogen atmosphere and room temperature for 4 h and then evaporated. After co-evaporation with toluene and dichloromethane, the crude mixture was separated by silica gel column chromatography using 20% ethyl acetate in dichloromethane as an eluent. Yield—278 mg. ^1^H-NMR (CDCl_3_): 10.10 (1H, s, aldehyde), 9.16 (1H, d, 2,4-dinitroaniline residue, *J* = 2.7 Hz), 8.59 (1H, m, –C(O)NH–), 8.29 (1H, dd, 2,4-dinitroaniline residue, ^2^*J* = 9.6 Hz, ^3^*J* = 2.7 Hz), 7.95–7.92 (4H, m, 1,4-phenylene), 6.93 (1H, d, 2,4-dinitroaniline residue, *J* = 9.6 Hz), 6.27 (1H, m, –NH–), 3.55–3.42 (4H, 2 × q, –C(O)NHCH_2_– and –CH_2_NH–), 1.87–1.49 (8H, m, –CH_2_CH_2_CH_2_CH_2_–).

### 2.6. Synthesis of Releasable HA-//-BP Conjugate

Thiol-modified hyaluronic acid HA-SH (40 mg, 0.1 mmol of disaccharide repeat units, 14 µmol of thiol groups) was dissolved in deionized water (5 mL) whereas reagent **4** (16.4 mg, 14 µmol) was dissolved in 1 mL of water. The aqueous solution of **4** was added into the HA-SH solution and pH of the combined solution was adjusted to 7.5 with 1M NaOH. The reaction was continued under stirring for 25 h. The mixture was transferred into a dialysis tube (M_w_ cutoff = 3500). After three dialysis rounds, firstly against acidified water (pH 4) containing 0.1 M NaCl (1 × 2 L) and then against acidified water (2 × 2 L), the solution was lyophilized to give 31 mg of HA-//-BP (yield—77.5%). The incorporation of BP groups via disulfide linker was verified by ^1^H-NMR.

### 2.7. Synthesis of Releasable BP-HA-//-DN Conjugate and Its Characterization by Dynamic Light Scattering (DLS)

Hyaluronic acid dually modified with bisphosphonate and thiol groups (BP-HA-SH) (38.7 mg, ~10 µmol of thiol groups) was dissolved in deionized water (4 mL) and pH of the obtained solution was nuetralized with 1M NaOH (60 µL). Reagent **5** (9.2 mg, 17 µmol) was dissolved in 0.5 mL of *N*-methylpyrrolidone (NMP) and the obtained solution was added to BP-HA-SH solution which resulted in precipitation of the reagent. To homogenize the mixture, 3.5 mL of NMP was added to the mixture and the cleared reaction solution was stirred at room temperature for 23 h. The mixture was transferred into a dialysis tube (M_w_ cutoff = 3500) and dialyzed initially against DMSO (1 × 200 mL) for 90 min and then against water (4 × 2 L) for overall period of two days. After filtration of the dialyzed solution through a cotton, it was obtained 39.8 mg of BP-HA-//-DN (yield—99.5%). The incorporation of BP groups via disulfide linker was verified by ^1^H-NMR.

### 2.8. Synthesis of hy-HA-//-BP Derivative

Hyaluronic acid dually modified with hydrazide and thiol groups (hy-HA-SH) (40 mg, 0.1 mmol of disaccharide repeat units, ~10 µmol of thiol groups and ~8 µmol of hydrazide groups) was dissolved in deionized water (5 mL). Reagent **4** (12.2 mg, 10 µmol) was dissolved in 750 µL of water and added to the solution of hy-HA-SH. pH of the combined solution was adjusted to 7.5 with 1M NaOH. The reaction solution was stirred for 22 h and then transferred into a dialysis tube (M_w_ cutoff = 3500) and dialyzed firstly against acidified water (pH 4) containing 0.1 M NaCl (1 × 2 L) and then against acidified water (2 × 2 L). After three dialysis rounds, the solution was freeze-dried to produce 38 mg of hy-HA-//-BP (yield—95%). The incorporation of BP and availability of intact hydrazide groups was verified by gel tests with CaCl_2_ and HA-al derivatively, respectively.

### 2.9. Synthesis of DN-hyd-HA-//-BP Derivative and Simvastatin Loading

hy-HA-//-BP derivative (20 mg, ~4 µmol of hydrazide groups) was dissolved in 1 mL of water and 1 mL of DMSO was added to the solution. The reagent **6** (4.8 mg, 10.1 µmol) was dissolved in 2 mL of DMSO and added to the solution of hy-HA-//-BP. The reaction was continued for the next 24 h. The reaction solution was transferred into a dialysis tube (M_w_ cutoff = 3500) and dialyzed against DMSO (2 × 200 mL and 1 × 100 mL) until no free reagent **6** was detected in the dialysate. The dialyzed against DMSO colorless solution (10 mL) was divided into two equal parts (2 × 5 mL). One part was directly transferred into a dialysis tube (M_w_ cutoff = 3500) and dialyzed against water (2 L, pH 7.4 adjusted with 1M NaOH). At the same time, 1 mg of simvastatin was dissolved in another part of the dialyzed DMSO solution and the solution was further dialyzed against water for 24 h. The dialysis was repeated for 4 h for the both SIM-loaded and non-loaded DN-*hyd*-HA-//-BP. The dialyzed against water turbid solutions were freeze-dried providing 9.1 mg of DN-*hyd*-HA-//-BP and 8.6 mg of SIM@DN-*hyd*-HA-//-BP.

### 2.10. Formation of HA-//-BP•Ca^2+^ Hydrogel and Its Thiol-Triggered Dissolution

Hydrogel samples of 200 µL by volume and concentrations of HA-//-BP and Ca^2+^ ions being 3% *w/v* and 0.2 M respectively were prepared. For this purpose, 175 µL solution of HA-//-BP with concentration 3.4% was prepared and neutralized with 1M NaOH and the solution was diluted with water until 185.4 µL. 14.6 µL of the 2.75 M CaCl_2_ solution was subsequently added to the neutralized HA-//-BP solution which resulted in immediate gel formation. The hydrogel was homogenized with a pipette tip.

### 2.11. Formation of DN-//-HA-BP•Ca^2+^ Hydrogel

3.3 mg of DN-//-HA-BP was dissolved within 15 min in 100 µL water providing 3.3% of a very viscous and opaque solution. 8 µL of the 2.75 M CaCl_2_ solution was added to DN-//-HA-BP solution which resulted in gel formation. For neutralization, 2 µL of 1M NaOH was added to the gel which was homogenized with a pipette tip. The final volume of the mixture was 110 µL and concentrations of DN-//-HA-BP and Ca^2+^ ions were 3% and 0.2 M, respectively.

### 2.12. Study of DN Release from DN-//-HA-BP•Ca^2+^ Hydrogel

DN-//-HA-BP**•**Ca^2+^ hydrogel with average mass of 100 mg was incubated in either 0.5 mL of 0.17 M NaCl solution containing 2.5 mM CaCl_2_ and 20 mM dithiothreitol (DTT) or 0.5 mL of 0.17 M NaCl solution containing 2.5 mM CaCl_2_. In 24 h the incubation media were replaced with the fresh ones of the same composition and the collected release media were analyzed by RP-HPLC using a gradient of buffer B from 0 to 100% over 30 min (buffer A: 50 mM triethylammonium acetate (TEAA), pH 6.5; buffer B: 50% MeCN in 50 mM TEAA, pH 6.5). The detection was done at 363 nm. Number of examined hydrogel samples was three per group (incubation with or without DTT).

### 2.13. Study of Simvastatin Release from SIM@DN-//-HA-BP•Ca^2+^ Nanogel

1.5 mg of SIM@DN-*hyd*-HA-//-BP was dissolved in 400 µL of 0.1 M NaOAc/AcOH pH = 5.0 buffer, whereas another 1.5 mg of SIM@DN-*hyd*-HA-//-BP was dissolved in 400 µL of 1 × PBS buffer pH = 7.4. The dissolved samples were transferred into dialysis bags with a 3500 Da cut off and dialyzed against 5 mL of the same buffer. The released media were collected at determined equal time intervals (24 h) and the dialysis bags were placed in fresh media (5 mL) of the same content and pH. The collected media were analyzed by RP-HPLC using a gradient of buffer B from 0 to 100% over 30 min followed by elution with 100% buffer B for 5 min (buffer A: 50 mM triethylammonium acetate (TEAA), pH 6.5; buffer B: 90% MeCN in 50 mM TEAA, pH 6.5). The detection was done at 246 nm. The experiments were performed in triplicate.

### 2.14. Statistical Analysis

All released data are presented as mean ± standard deviation (SD). Each release experiment was repeated at least three times. Statistical analysis was performed using the statistical software Origin 6.1.

## 3. Results and Discussion

### 3.1. Synthesis of Releasable HA-//-BP Conjugate

Previously, we prepared hyaluronan (HA) derivatives in which aminobisphosphonates were permanently attached to HA backbone through stable chemical bonds, thereby excluding possibility for the release of aminobisphosphonates as intact drugs [29,30]. It was also demonstrated that these HA-BP derivatives formed shear-thinning and self-healing hydrogels upon interaction with either hydrated metal ions such as Ca^2+^ [31], Mg^2+^ [32], Ag^+^ [33], or nanoparticles of inorganic salts such as calcium phosphate [30], magnesium silicate [32], calcium sodium phosphosilicate (bioglass) [34], and clay [35].

**Scheme 1:**
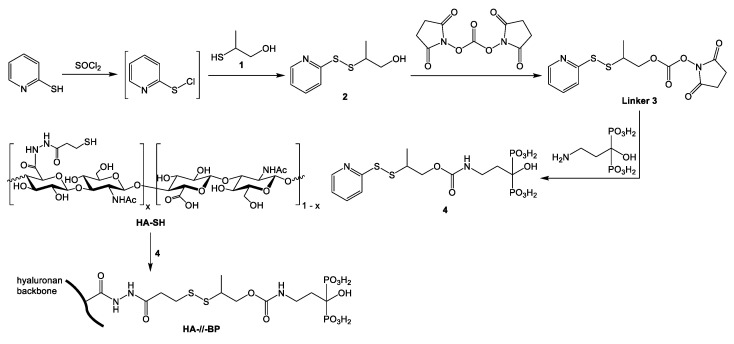
Synthesis of heterobifunctional linker **3** and hyaluronan-bisphosphonate prodrug HA-//-BP with a thiol-triggered release mechanism.

In this work, we utilized a prodrug approach exploiting drug release after cellular uptake and entering into cytoplasm where the concentration of bio-thiols such as glutathione is almost one thousand times higher than in the blood plasma. To prepare bisphosphonate prodrug via its conjugation to HA carrier, we designed a linker that has terminals that are orthogonally reactive towards amines and thiols (Scheme 1). Starting with 1-methyl-2-hydroxyethylthiol **1** and 2-thiopyridine, we synthesized 2-dithiopyridyl compound **2,** following activation of the hydroxyl with di-*N*-succinimidylcarbonate provided linker **3** containing 2-dithiopyridyl group on one end and *N*-succinimidylcarbonate group on the other end of the linker. Drug pamidronate was then coupled to the linker through a carbamate linkage, which gave bisphosphonate derivative **4** that is amenable to further disulfide coupling with molecules carrying free thiol groups. Compound **4** was correspondingly linked to hyaluronic acid modified with thiol groups (HA-SH).

Thus, a new hyaluronan-bisphosphonate prodrug HA-//-BP with a thiol-triggered release mechanism was obtained. Previously, low molecular weight aminobisphosphonate prodrugs were prepared to be responsive to enzymatic hydrolysis of phenol carbamates [22] or cleavage of amide bond by cathepsin K [23]. Alternatively, lipophilic pivaloyloxymethyl esters of bisphosphonates were shown to be converted to active bisphosphonates by intracellular esterases [36]. In this work, a polymeric bisphosphonate prodrug activatable by biothiols in cytoplasm was prepared first. A sterically hindered disulfide bond in the middle of the releasable linker was chosen purposefully to increase the stability of the disulfide bond and ensure the drug attachment during circulation in the blood [24]. It should be noted, however, that formation of sterically hindered linear disulfide is also expected to be slower in comparison with the formation of unhindered disulfide. Despite this limitation, the successful attachment of BP to HA was confirmed by UV–Vis spectrophotometric observation of the generation of 2-thiopyridine side-product of the reaction (Figure S1) as well as by ^1^H- and ^31^P-NMR spectroscopy (Figure S2). A characteristic peak at 2.14 ppm corresponding to protons of methylene group adjacent to the bridging bisphosphonate carbon was observed. Another characteristic peak of methyl substituent adjacent to disulfide bond was also observed at 1.26 ppm. A peak at 4.12 ppm was attributed to the methylene protons neighboring the carbamate oxygen atom of the linker and the rest of the protons of the linker were grouped in the region between 2.7 and 3.2 ppm (except for the methylene protons near the carbamate NH group which were overlapped by HA saccharide).

A self-immolative 2-methylethoxycarbonyl fragment between the disulfide bond and the amino group of pamidronate (designated as -//-) was designed to provide intact release of pamidronate molecules and, thus, the activation of the drug after cleavage of the disulfide bond in the cytoplasm. Previous polymer-based prodrugs exploited only glutathione-cleavable disulfanyl ethylcarbonate self-immolative side chains, which are sterically more accessible and thus should be less stable in the extracellular space [37,38]. Finally, we used HA polymeric material with multiple presentation of BP groups to engage them in coordination bonding to metal ions as well as to impart bone-adhesive properties to the corresponding coordination hydrogels.

### 3.2. Coordination Hydrogel Formed by Releasable HA-//-BP

**Figure 2:**
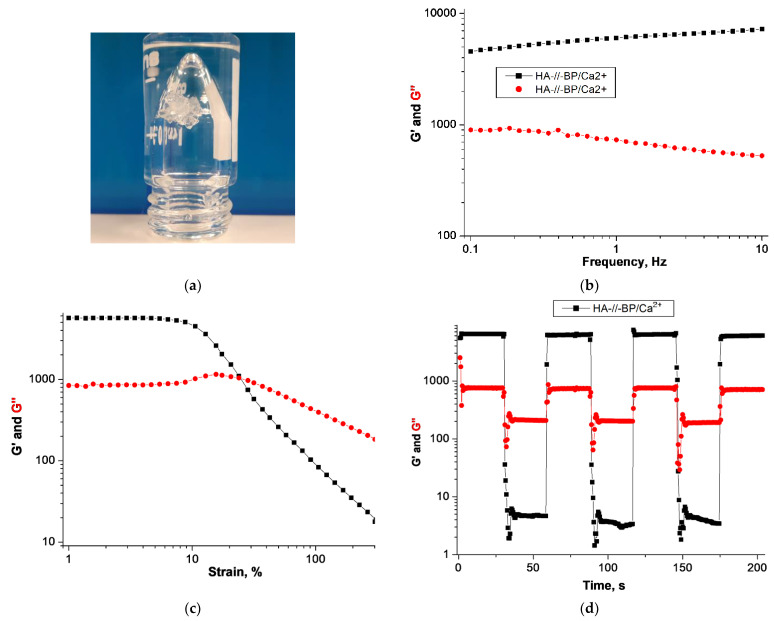
(**a**) Image of hydrogel formed by coordination cross-linking of HA-//-BP derivative by Ca^2+^ ions. (**b**) Frequency oscillation sweep of HA-//-BP•Ca^2+^ hydrogel. (**c**) Strain oscillation sweep of HA-//-BP•Ca^2+^ hydrogel. (**d**) Time oscillation sweep of HA-//-BP•Ca^2+^ hydrogel upon alteration of strain between low (1%) and high (300%) values. Concentrations of HA-//-BP and Ca^2+^ ions in HA-//-BP•Ca^2+^ hydrogel were 3% and 0.2 M, respectively.

Releasable HA-//-BP derivative readily formed a hydrogel upon mixing with a solution of CaCl_2_ (Figure 2a). HA-//-BP**•**Ca^2+^ hydrogel is an example of macromolecular network in which macromolecular chains are cross-linked through metal ions (i.e., on a molecular level). The viscoelastic properties of the hydrogel were examined in a frequency oscillation sweep experiment (Figure 2b). Moreover, an increase of strain from 1 to 300% during the strain oscillation sweep indicated a decrease of storage (G′) modulus and an increase of loss (G′′) modulus starting from a strain value of 10% and reaching a crossover point at already ~25% strain (Figure 2c). This emphasizes the shear-thinning properties of the hydrogel. The formed hydrogel clearly displayed self-healing properties, as was judged from the time oscillation sweep experiment consisting of four cycles of alteration of strain between low (1%) and high (300%) values (Figure 2d). It implies that hydrogels formed by releasable HA-//-BP derivative and Ca^2+^ ions are fully injectable and can recover their mechanical properties autonomously after injection.

We tested the ability of the new HA-//-BP**•**Ca^2+^ hydrogel to respond to thiols. Cleavage of disulfide bond in the linker connecting BPs with an HA backbone should result in the hydrogel dissolution and the release of any cargo molecules that are physically entrapped in the hydrogel. Moreover, such hydrogel dissolution should convert the hydrogel-linked BP prodrug into the actual bisphosphonate drug. To prove this, we incubated HA-//-BP**•**Ca^2+^ hydrogel in 0.17 M NaCl containing 2.5 mM CaCl_2_ and 20 mM dithiothreitol (DTT) at pH 7.5. The hydrogel formed by HA-//-BP derivative was almost completely decomposed after 30 min of incubation and, after 1 h of incubation, a white precipitate was formed in place of the hydrogel. The solid material formed upon interaction of DTT with HA-//-BP**•**Ca^2+^ hydrogel is the insoluble calcium pamidronate which should be generated after the detachment of pamidronate from the network. Additionally, we examined previously reported HA-BP analog [30] characterized by permanent attachment of BP groups (see structure in Figure S3 in Supplementary Materials). As expected, HA-BP**•**Ca^2+^ hydrogel stayed intact even after 3 days of incubation with DTT.

### 3.3. Synthesis and Characterization of Releasable BP-HA-//-DN Conjugate

**Scheme 2:**
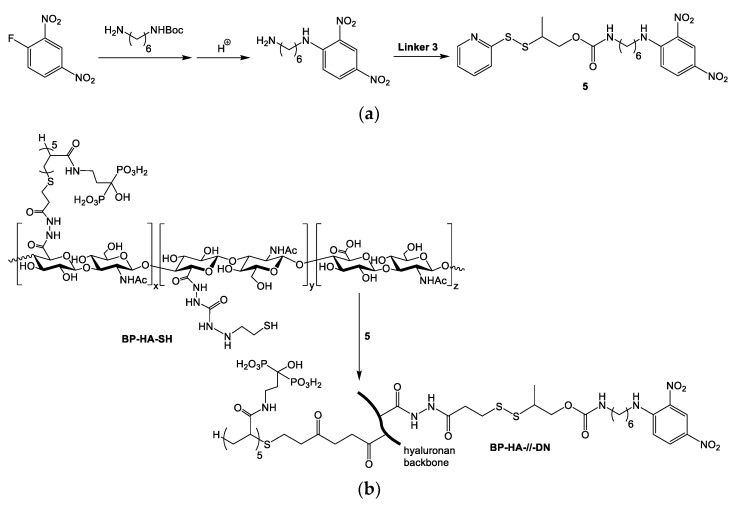
(**a**) Synthesis of the model prodrug **5** carrying 2-dithiopyridyl groups and (**b**) its further conjugation to hyaluronan to obtain HA derivative with permanently attached BP groups and releasable prodrug moieties (BP-HA-//-DN).

To study the thiol-triggered drug release, we prepared an HA derivative in which BP groups were permanently attached to the HA backbone whereas another hydrophobic model amine was linked to HA via releasable linker **3**. Specifically, *N*-(6-aminohexyl)-2,4-dinitroaniline (DN) was coupled to linker **3** via carbamate bond (Scheme 2a) and the resulting compound **5** was conjugated to HA derivative modified with BP and thiol groups (BP-HA-SH) (Scheme 2b). BP-HA-SH derivative was prepared as previously described [28] and it is characterized by permanent attachment of BP groups to the HA backbone.

Due to the hydrophobic properties of the attached ligand, the obtained BP-HA-//-DN derivative underwent self-association in the aqueous medium, forming nanogel particles with a hydrophobic DN core and hydrophilic HA-BP shell. The formation of nanogels was confirmed by dynamic light scattering measurements (Figure S4a). The size of the formed particles was ~513 nm. Similar nanogels formation was reported for HA dually modified with hydrophobic 5*β*-cholanic acid and metal ion-chelating dipicolylamine groups [39]. The size of the reported nanogels was around 225 nm, which is less than twice the size of the newly prepared nanogels. It should be noted that the size of hydrohpobically self-assembled nanogels is dependent on many factors including the nature of appended groups, molecular weight of a backbone polymer, and the degrees of modification with the side chains.

The particles were subsequently treated with dithiothreitol (DTT) to cleave the disulfide bond between the HA backbone and hydrophobic DN moieties and, hence, to destroy the particles. According to DLS measurements, it led to the decrease of the particles size to ~388 nm (Figure S4b) and the decrease of the particles’ concentration six times (Figure S4c). These results can be rationalized by the hydrophobicity of the released DN molecules which tend to aggregate after cleavage, thus preventing the complete nanoparticles disappearance in the dispersion.

### 3.4. Thiol-Triggered Release of Covalently Linked Drugs

**Figure 3:**
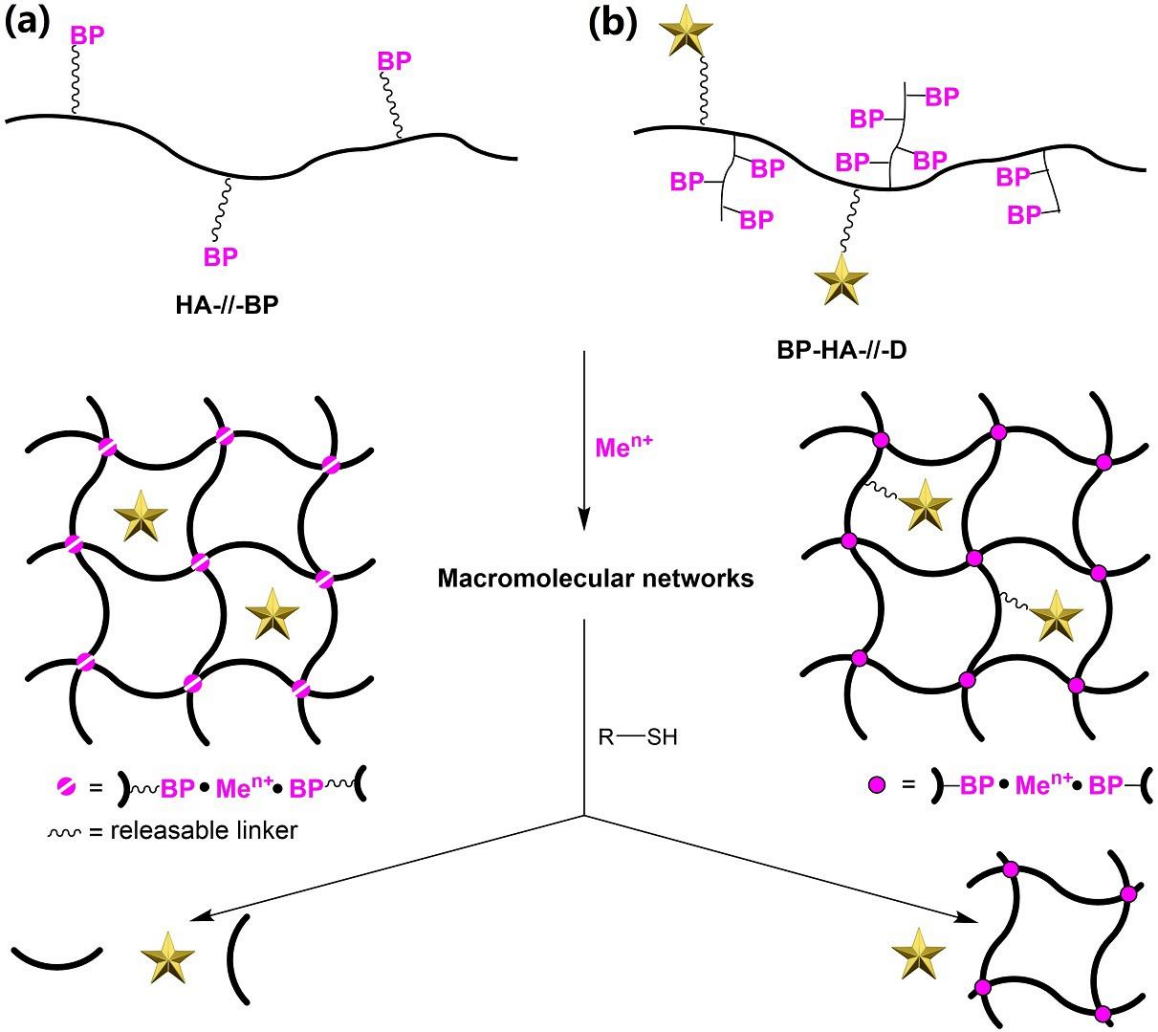
Two types of hyaluronan-bisphosphonate derivatives (HA-//-BP and BP-HA-//-D where D stands for a drug and -//- stands for a releasable self-immolative linker) and the respective injectable hydrogel-based drug delivery systems: (**a**) Intact bisphosphonate-releasing hydrogel that is decomposed upon action of thiols and (**b**) hydrogel-linked prodrug that releases amine-bearing drugs upon action of thiols.

In this work, we anticipated two hydrogel-based drug delivery approaches. In one approach accomplished with the HA-//-BP derivative, the cleavable linkers are placed between HA backbone and chelating BP groups (Figure 3a). The reduction of disulfide bonds in a hydrogel formed by coordination of HA-//-BP to metal ions should lead to the hydrogel dissolution and the release of physically encapsulated cargo molecules. At the same time, free aminobisphosphonate molecules are generated. In the second approach accomplished with the BP-HA-//-DN derivative, the releasable linker is placed between a biopolymer and some relevant drug (DN in this case), whereas BP ligands are permanently conjugated to HA (Figure 3b). Therefore, thiols do not affect the corresponding coordination hydrogel, but instead trigger release of a drug from the hydrogel.

**Figure 4:**
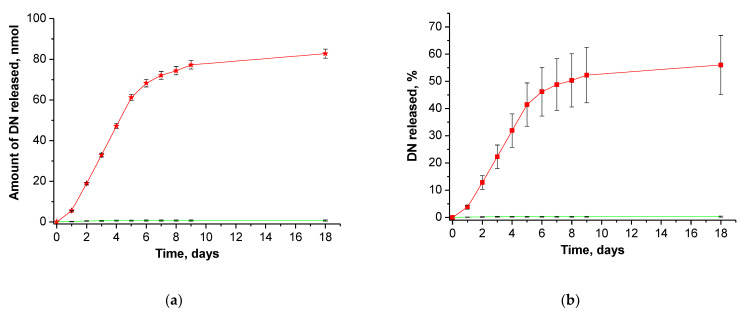
(**a**) Absolute amount and (**b**) percentage of *N*-(6-aminohexyl)-2,4-dinitroaniline (DN) released from Ca^2+^•BP-HA-//-DN hydrogel upon incubation in 0.17 M NaCl containing 2.5 mM CaCl_2_ (pH 7.4). The red curve corresponds to the release when 20 mM DTT was present in the incubation medium. The green curve corresponds to the release when no DTT was present in the release medium.

The ability of the synthesized macromolecular prodrug BP-HA-//-DN to form self-healing macromolecular or colloidal hydrogels was subsequently evaluated. The hydrogels were readily formed, and they were both characterized by self-healing properties (Figure S5). The Ca^2+^•BP-HA-//-DN hydrogel was subsequently incubated in an aqueous buffer at pH 7.4. Alternatively, the incubation was performed in the buffer containing 20 mM dithiothreitol (DTT). The results of the release studies are shown in Figure 4. We observed almost no release of the model DN drug under non-reducing conditions. However, the addition of DTT initiated the release of DN which occurred almost at a constant rate between the second and fifth day. After six days, the release was gradually diminished.

To the best of our knowledge, the demonstrated model drug release from HA hydrogel is the first study of a hydrogel-linked prodrug approach which exploits the thiol-disulfide exchange reaction in the interior of a macroscopic hydrogel. Mainly, disulfide bonds were introduced as part of cross-linkages between polymeric chains [40], thus enabling the thiol-triggered release of encapsulated molecules or cells through the hydrogel’s dissolution (as exemplified also in Figure 3a). The use of disulfide bonds as side linkages of differently cross-linked networks was so far utilized only for demonstration of modular “catch and release” from a hydrogel surface [41].

### 3.5. Synthesis of DN-hyd-HA-//-BP Conjugate with Dual Release Mechanism

For the delivery of aminobisphosphonates and physically encapsulated hydrophobic simvastatin, we anticipated the orthogonal conjugation of hydrophilic BP and hydrophobic DN ligands through differentially cleavable linkers. Specifically, we sought to achieve hydrolytic cleavage of DN ligands under acidic conditions while initiating the release of BP drugs in the reducing microenvironment of cytoplasm. Release under slightly acidic conditions was rationalized by two reasons. Thus, tumor microenvironments are characterized by slightly acidic pH (around 6.8), as well as bone resorption occurs because of the acidic dissolution of calcium phosphate mineral. On the other hand, the uptake of the HA-based nanogels by HA receptor-mediated endocytosis should bring them into endosomes and later to lysosomes, which are characterized by microenvironments with gradually decreasing pH from 6.8 to 5.

**Scheme 3:**
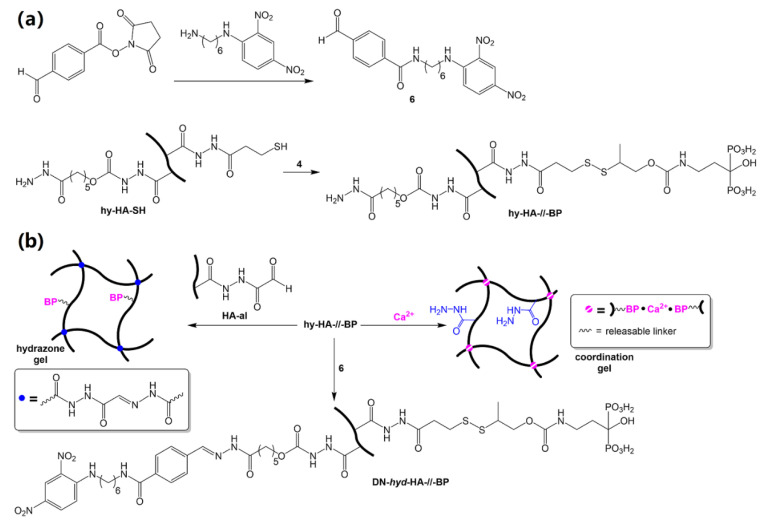
(**a**) Synthesis of aldehyde-modified DN derivative and hyaluronic acid bearing hydrazide and releasable BP groups (hy-HA-//-BP). (**b**) Use of hy-HA-//-BP derivative for the preparation of hydrazone and coordination hydrogels as well as for generation of amphiphilic HA derivative bearing hydrophobic DN and hydrophilic BP groups (DN-hyd-HA-//-BP).

Based on the above considerations, DN-*hyd*-HA-//-BP derivative (hyd denotes hydrazone linkage, whereas -//- denotes self-immolative disulfide linkage) was prepared starting from hyaluronic acid modified with hydrazide and thiol groups (hy-HA-SH) (Scheme 3a). Dually modified HA was first treated with BP reagent **4** for 24 h in an aqueous solution at pH 7.5 providing the intermediate hy-HA-//-BP carrying hydrazide and BP groups.

The functionality of the hydrazide and BP groups in hy-HA-//-BP was verified by gel tests. Particularly, hydrazone cross-linked and coordination hydrogels were successfully formed upon the mixing of hy-HA-//-BP with aldehyde-modified HA (HA-al) and CaCl_2_ solutions (Scheme 3b). The treatment of hy-HA-//-BP with aldehyde-modified DN derivative **6** was subsequently performed in a mixture of water and DMSO (1:3, *v/v*) at pH 5 for 24 h. The excess of unreacted **6** was removed by repeated dialysis of the reaction mixture against DMSO which was verified by UV–Vis spectroscopy (Figure S6). Final dialysis against water provided self-assembly of the amphiphilic DN-hyd-HA-//-BP derivative (Scheme 3b) which was verified by DLS analysis (Figure S7a).

**Figure 5:**
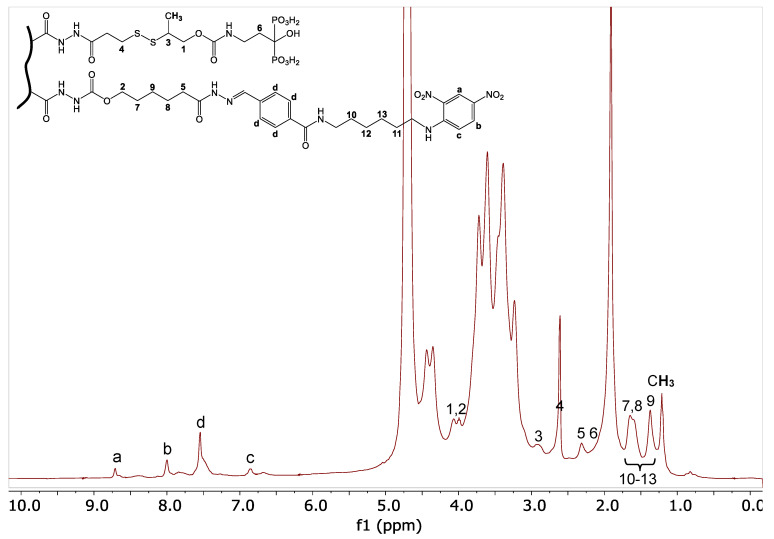
^1^H-NMR spectrum of DN-hyd-HA-//-BP derivative.

The obtained DN-hyd-HA-//-BP was characterized by ^1^H-NMR spectroscopy (Figure 5). Aromatic protons of 2,4-dinitroaniline and phenylene moieties were observed at 8.7, 8.0, 7.5, and 6.8 ppm. It confirmed the hydrazone coupling of reagent **6** to hy-HA-//-BP. The successful disulfide attachment of BP derivative **4** was evident from the appearance of the peak of methyl substituent of **4** at 1.2 ppm. Other peaks corresponding to methylene groups of the precursor compounds **4** and **6** (designated in Figure 5 as 1, 2, 4, and 5–13) were also identified in the spectrum.

To evaluate the possibility of acidic release of physically loaded cargos, we encapsulated simvastatin (SIM) in DN-hyd-HA-//-BP nanogels. Simvastatin was added to DN-hyd-HA-//-BP in DMSO and subsequently dialyzed against water to permit homogeneous encapsulation of SIM in DN-hyd-HA-//-BP nanogels. Dialysis of the mixture against water provided substitution of DMSO to water and the entrapment of SIM in the hydrophobic pocket formed by the self-assembled DN moieties. The hydrodynamic diameter of SIM loaded nanogels was not altered much in comparison with the unloaded DN-hyd-HA-//-BP nanogels, as was verified from DLS measurements (Figure S7b). The SIM encapsulation efficiency was estimated by UV–Vis spectroscopy and found to be 55 µg/mg of SIM@DN-hyd-HA-//-BP, which corresponded to a loading efficiency of 47.3%. This loading efficiency was four times higher than the reported encapsulation of SIM in HA nanogels prepared by disulfide cross-linking of 11-amino-1-undecanethiol hydrophobic side chains attached to the HA backbone [42].

### 3.6. Acid-Triggered Release of DN Imaging Groups and Physically Encapsulated Simvastatin

**Figure 6:**
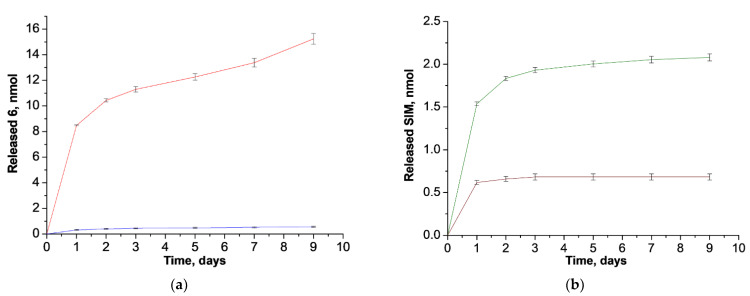
(**a**) Amount of DN derivative 6 released upon incubation of SIM@DN-*hyd*-HA-//-BP in either 0.1 M NaOAc/AcOH pH = 5.0 buffer (red curve) or 1 × PBS buffer pH = 7.4 (blue curve). (**b**) Amount of simvastatin (SIM) released upon incubation of SIM@DN-*hyd*-HA-//-BP in either 0.1 M NaOAc/AcOH pH = 7.4 buffer (green curve) or 1 × PBS buffer pH = 7.4 (brown curve).

The pH-controlled release of DN reagent **6** from SIM@DN-*hyd*-HA-//-BP was investigated (Figure 6a). As expected, the detachment of hydrophobic DN moieties was accompanied by the release of the physically encapsulated SIM (Figure 6b). This result was expected because the acidic hydrolysis of the hydrazone bond between hydrophobic DN residues and hydrophilic HA backbone should disassemble the nanogel particles. The release of both **6** and SIM occurred largely during the first two days, but with much less burst effect than is normally observed from hydrogels physically loaded with SIM [43]. We noticed that the release of SIM was diminished in comparison with the release of **6**. HPLC analysis revealed that SIM is substantially more hydrophobic than **6,** which anticipates less solubility of SIM in the aqueous media. SIM can be effectively dispersed in water when entrapped in DN-*hyd*-HA-//-BP nanogels. However, disassembly of the nanogels should also be accompanied by aggregation/precipitation of SIM.

Essentially, the release of SIM from nanogels particles becomes halted due to the transformation of the system of homogeneously dispersed nanogels into a heterogeneous two-phase system. This limitation of the present in vitro system might be overcoming by concerted hydrolytic and enzymatic HA degradation in vivo. Nonetheless, the current in vitro model demonstrates that the release of SIM is pH-controlled if the drug is hydrophobically encapsulated in an amphiphilic conjugate in which hydrophobic and hydrophilic parts are linked through a hydrazone bond.

The prepared DN-*hyd*-HA-//-BP derivative is multifunctional because it can form both bulk self-healing hydrogels and nanogels, depending on the functionalized biopolymer concentration. These different hydrogel formulations can be used interchangeably as localized drug depots and for the systemic delivery of drugs, respectively. Importantly, the developed hydrogel system is made multi-responsive by the combination of orthogonally cleavable linkers for the network-linked prodrug construction and for the attachment of moieties that enable hydrophobic encapsulation through self-association. In particular, the DN-*hyd*-HA-//-BP derivative enables the immobilization of hydrophilic aminobisphosphonates and hydrophobic simvastatin with their subsequent orthogonal and intact release upon the action of bio-thiols and acids, respectively.

## 4. Conclusions

In summary, we utilized different compartments of bisphosphonate-decorated nanogel particles and different types of labile chemical bonds to achieve the orthogonal release of pamidronate and simvastatin characterized by opposing solubility in aqueous media. Aminobisphosphonate pamidronate was conjugated to hyaluronic acid through a self-immolative disulfide linker, whereas hydrophobic imaging ligand (DN) was linked through an acid-labile hydrazone bond. The resulting amphiphilic DN-*hyd*-HA-//-BP conjugate was self-assembled into nanogel particles in aqueous media. We demonstrated that hydrophobic simvastatin can be physically entrapped in DN-*hyd*-HA-//-BP and subsequently released under acidic conditions, which was attributed to the hydrolysis of the hydrazone bond between DN residues and HA backbone. When DN residues were linked through the disulfide bond, as in the BP-HA-//-DN derivative, orthogonal cleavage under reducing conditions was observed. Moreover, various chemical attachments of BP ligands to HA biopolymer provided the possibility to form self-healing macroscopic hydrogels that deliver drugs either through stimuli-responsive hydrogel dissolution or chemical detachment from the network. This simple and modular approach provides injectable hydrogel depots which can release drugs locally in bone resorption sites. Alternatively, the prepared nanogels are potential candidates to deliver antiresorptive/anti-cancer and bone growth inducing drugs systemically to osteoporosis sites.

## Data Availability

All of the data are displayed in the article.

## Supplementary Materials

The following are available online at https://www.mdpi.com/article/10.3390/polym13132106/s1. Figure S1: UV–Vis characterization of the reaction of conjugation of bisphosphonate reagent **4** to thiolated HA (HA-SH); Figure S2: ^1^H- and ^31^P-NMR spectra for releasable HA-//-BP conjugate; Figure S3: Chemical structure of HA-BP derivative; Figure S4: (**a**) DLS of BP-HA-//-DN solution in water (0.125 mg/mL) (**b**) DLS of aqueous solution of BP-HA-//-DN treated with 30 mM DTT. (**c**) Diagram of particles concentration as a function of particles size; Figure S5: (**a**) Frequency oscillation sweep of Ca^2+^**•**BP-HA-//-DN hydrogel. (**b**) Frequency oscillation sweep of Ca^2+^**•**BP-HA-//-DN hydrogel. (**c**) Time oscillation sweep of Ca^2+^**•**BP-HA-//-DN; Figure S6: UV–Vis analysis of DMSO media used for the dialysis of reaction mixture of hydrazone coupling of **6** to hy-HA-//-BP derivative; Figure S7: (**a**) DLS of DN-*hyd*-HA-//-BP solution in water (0.5 mg/mL) and (**b**) DLS of SIM@DN-*hyd*-HA-//-BP solution in water (0.5 mg/mL).
