# Supplementary material for: Differentiating Co-Delivery of Bisphosphonate and Simvastatin by Self-Healing Hyaluronan Hydrogel Formed by Orthogonal “Clicks”: An In-Vitro Assessment
Source: Polymers (Basel). 2021 Jun 26;13(13):2106. doi: 10.3390/polym13132106 (PMC8272211; doi:10.3390/polym13132106)
Supplement: Supplementary file 1 [file polymers-13-02106-s001.zip › polymers-1268475-supplementary.pdf]

Differentiating Co-Delivery of Bisphosphonate and Simvastatin by Self-Healing Hyaluronan Hydrogel Formed by Orthogonal "Clicks": An In-Vitro Assessment

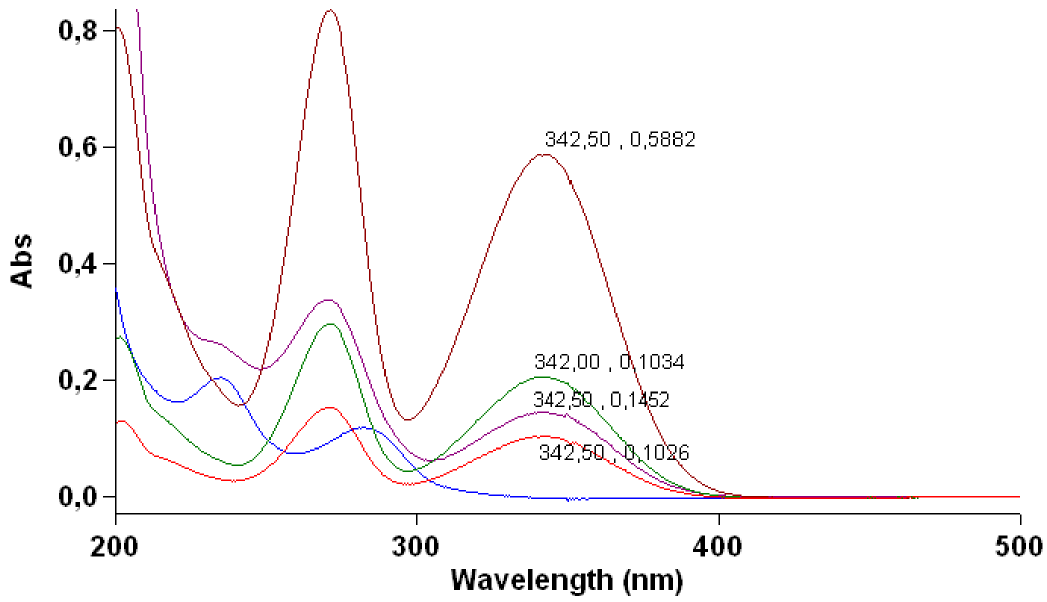

Figure S1. UV-Vis characterization of the reaction of conjugation of bisphosphonate reagent **4** to thiolated HA (HA-SH). Brown, green, and red curves correspond to authentic 2-thiopyridine samples with concentration of 100 µM, 35 µM, and 17.5 µM respectively. Blue curve corresponds to the solution of reagent **4** at 10 µg/mL concentration and purple curve correspond to a 20 µL aliquot taken from 6 mL of the reaction mixture of HA-SH with reagent **4**. The appearance of the peak at 342 nm in the reaction mixture indicates the generation of 2-thiopyridine as a side product of conjugation of reagent **4** to the HA backbone via disulfide linkage.

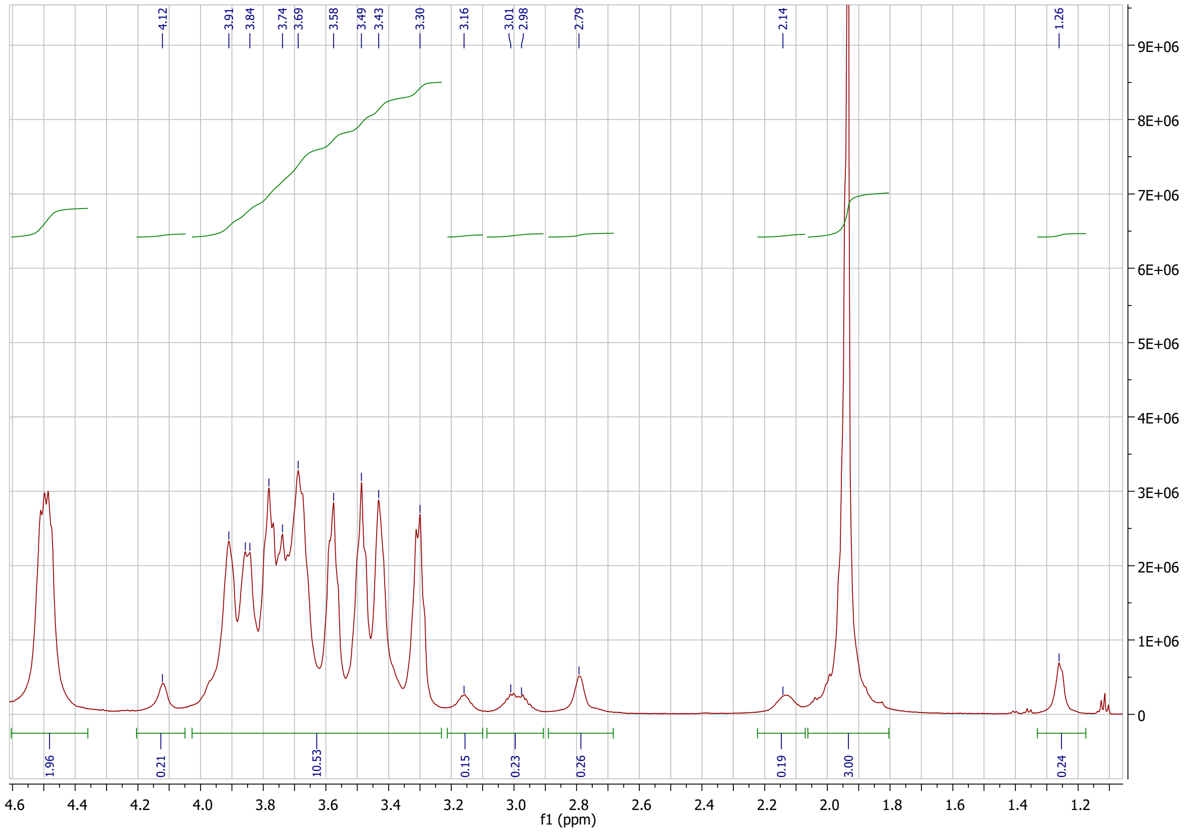

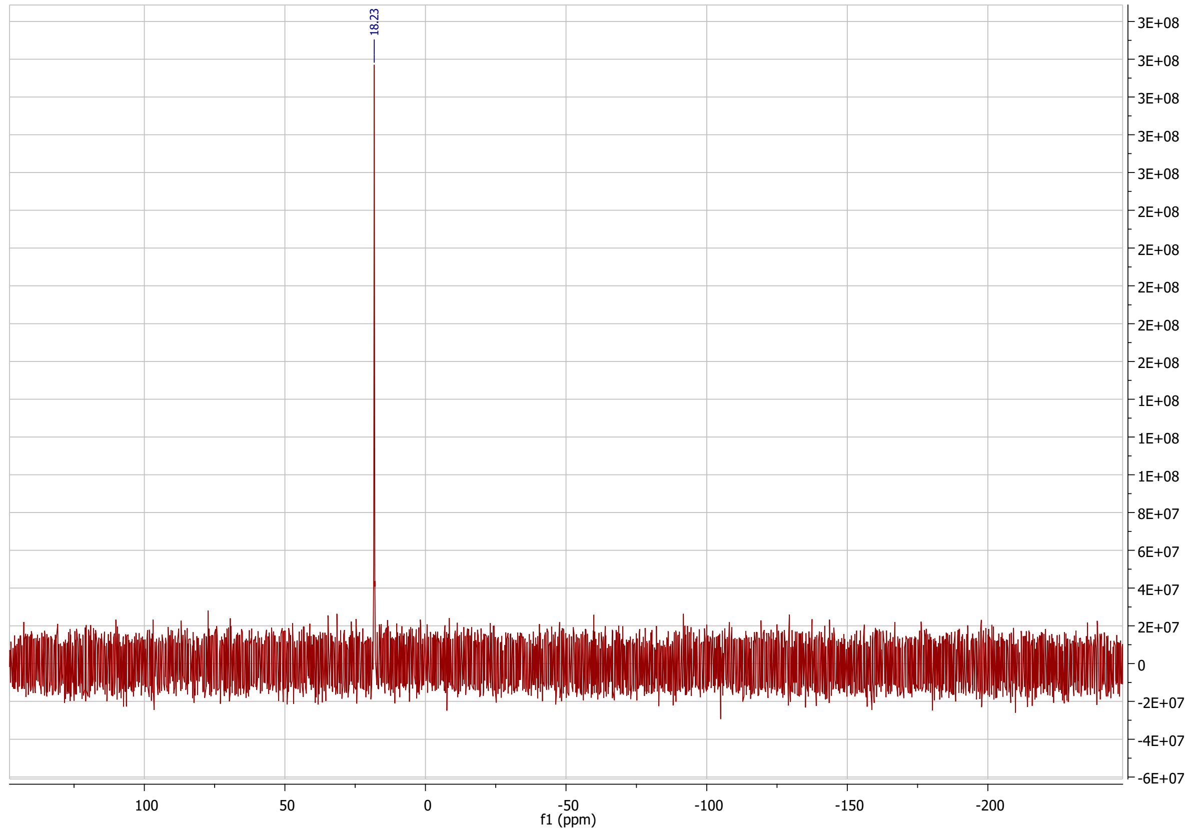

Figure S2. ^{1}H- and ^{31}P-NMR spectra for releasable HA-//-BP conjugate (polymeric BP prodrug).

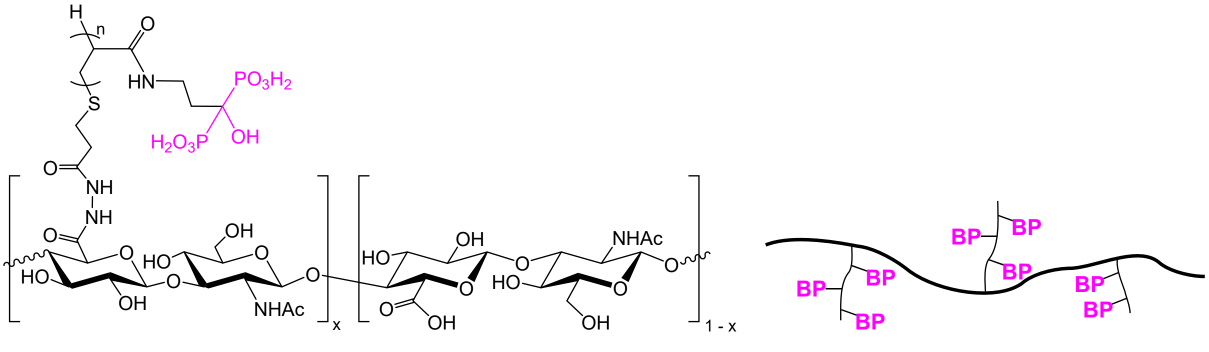

HA-BP

Figure S3. Chemical structure of HA-BP derivative and its schematic showing brushes of BP groups attached to a single monomer unit of HA macromolecule.

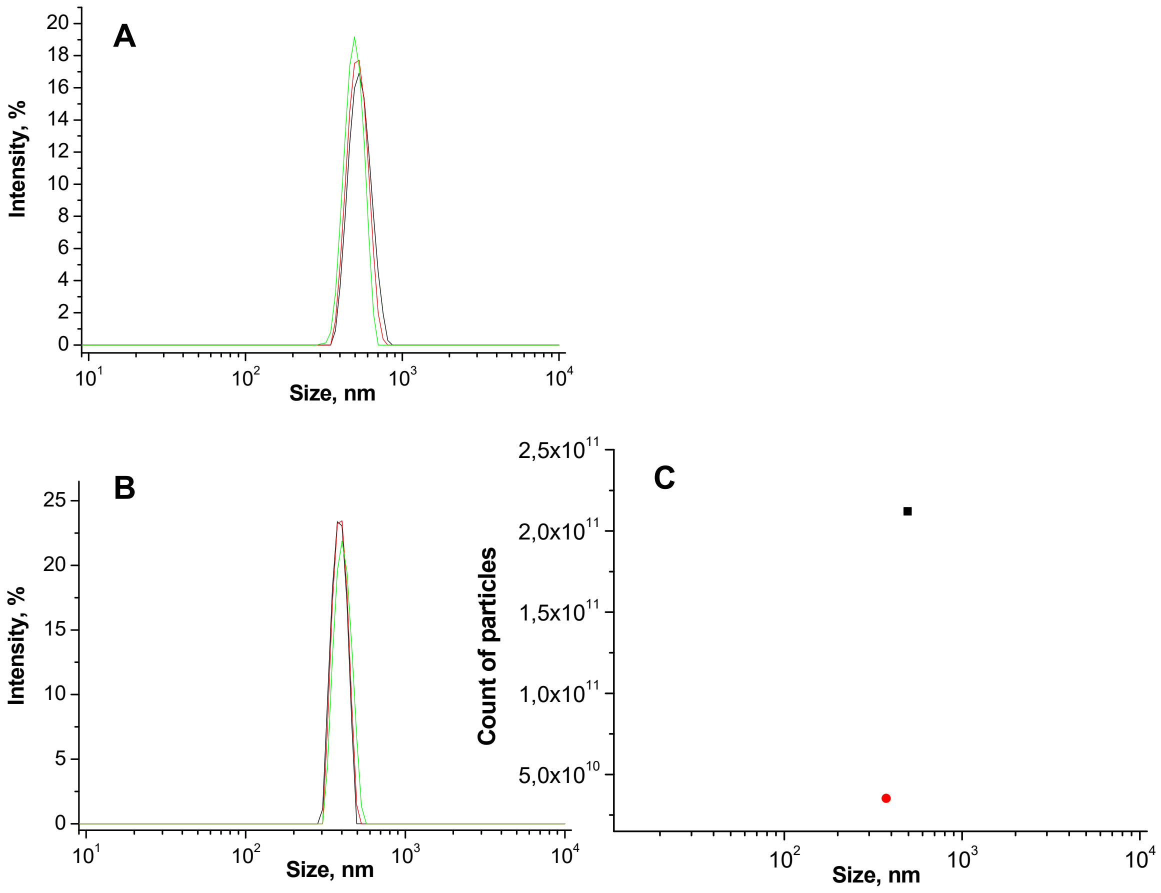

Figure S4. (a) DLS of BP-HA-//-DN solution in water (0.125 mg/mL) (b) DLS of aqueous solution of BP-HA-//-DN treated with 30 mM DTT. (c) Diagram of particles concentration as a function of particles size. Black and red dots correspond to the particles before and after treatment with DTT, respectively.

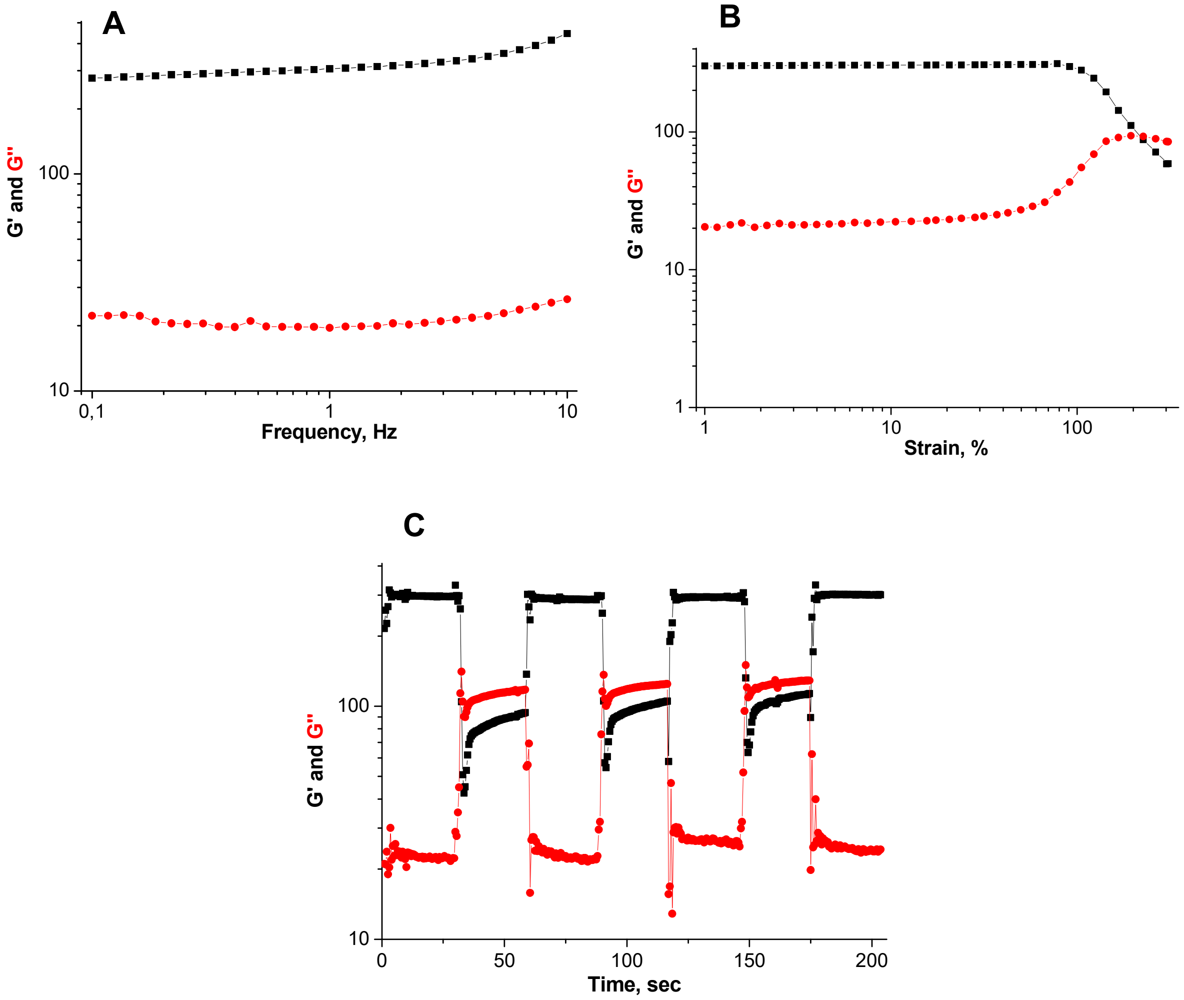

Figure S5. (a) Frequency oscillation sweep of Ca^{2+}•BP-HA-//-DN hydrogel. (b) Frequency oscillation sweep of Ca^{2+}•BP-HA-//-DN hydrogel. (c) Time oscillation sweep of Ca^{2+}•BP-HA-//-DN.

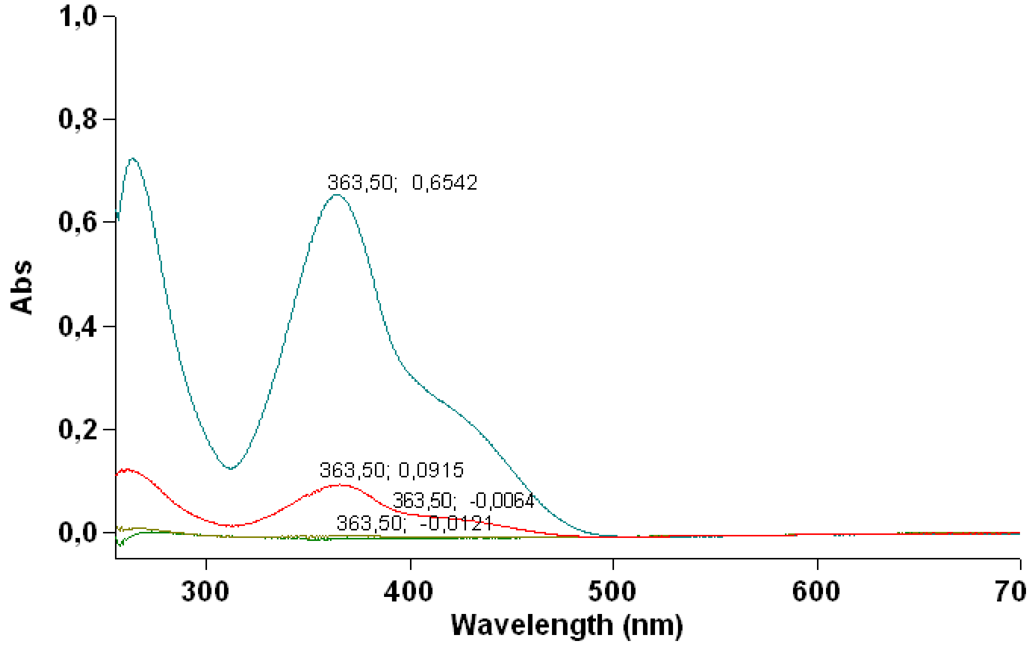

Figure S6. UV-Vis analysis of DMSO media used for the dialysis of reaction mixture of hydrazone coupling of **6** to hy-HA-//-BP derivative. 1 mL aliquots were taken from the 200 mL of DMSO media and analyzed by UV-Vis spectroscopy. The blue curve corresponds to the first dialysis round for 24 hours, the red curve corresponds to the second round. The third and fourth dialysis rounds showed no unreacted **6** in the respective DMSO media.

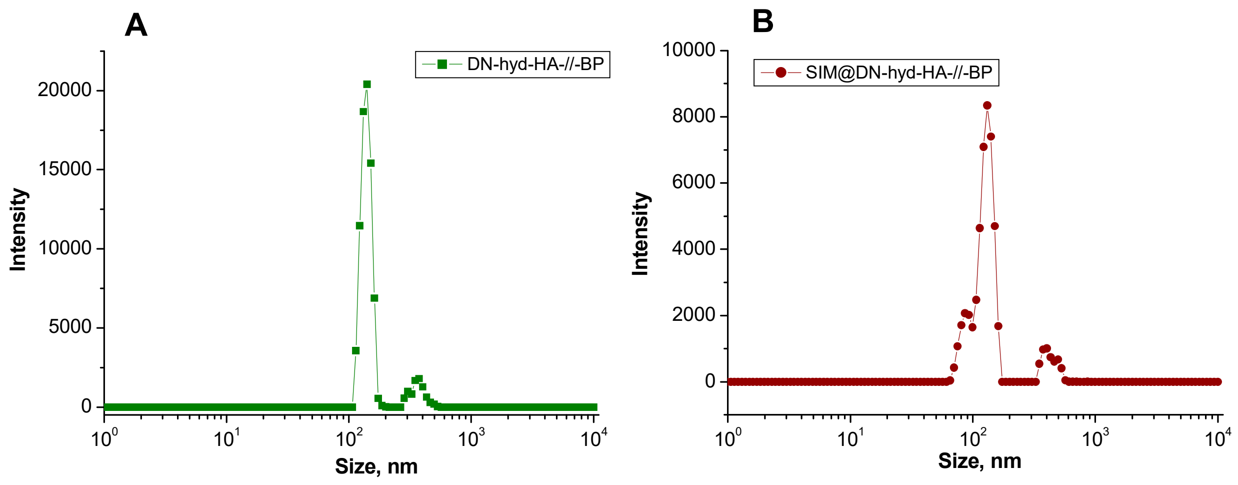

Figure S7. (a) DLS of DN-*hyd*-HA-//-BP solution in water (0.5 mg/mL) and (b) DLS of SIM@DN-*hyd*-HA-//-BP solution in water (0.5 mg/mL).

Investigation of responsiveness of different calcium ions-induced hyaluronan-bisphosphonate hydrogels to external thiols.

3.3 mg of HA-//-BP was dissolved within 20 min in 100 µL water providing 3.3% solution. This solution was neutralized with 5 µL of 1M NaOH. 8 µL of the 2.75 M $CaCl_2$ solution was added to the neutralized HA-BP solution which resulted in gel formation after a minute. The final volume of the gel was 113 µL and concentrations of HA-//-BP and Ca^{2+} ions were 3%

and 0.2 M respectively. The gel was set for 24 hours. After setting, the hydrogel was incubated in 2 mL 0.17 M NaCl solution containing 2.5 mM $CaCl_2$ and 20 mM dithiothreitol (DTT) (9.9 mg of DTT was dissolved in 3210 µL of 0.17 M NaCl and 2.5 mM $CaCl_2$).

For comparison, 3.3 mg of HA-BP was dissolved within 20 min in 95 µL water providing 3.3% solution. This solution was neutralized with 5 µL of 1M NaOH. 8 µL of the 2.75 M $CaCl_2$ solution was added to the neutralized HA-BP solution which resulted in immediate gel formation. The final volume of the gel was 108 µL and concentrations of HA-BP and Ca^{2+} ions were 3% and 0.2 M respectively. The gel was set for 24 hours. After setting, the hydrogel was incubated in 2.7 mL 0.17 M NaCl solution containing 2.5 mM $CaCl_2$ and 20 mM dithiothreitol (DTT) (8.5 mg of DTT was dissolved in 2755 µL of 0.17 M NaCl and 2.5 mM $CaCl_2$).
